# A novel *PAX7* 10-bp indel variant modulates promoter activity, gene expression and contributes to different phenotypes of Chinese cattle

**DOI:** 10.1038/s41598-018-20177-8

**Published:** 2018-01-29

**Authors:** Yao Xu, Tao Shi, Yang Zhou, Mei Liu, Sebastian Klaus, Xianyong Lan, Chuzhao Lei, Hong Chen

**Affiliations:** 1 College of Animal Science and Technology, Northwest A & F University, Shaanxi Key Laboratory of Molecular Biology for Agriculture, Yangling, Shaanxi 712100 China; 2 Institute of Biology and Medicine, College of Life Science and Health, Wuhan University of Science and Technology, Wuhan, Hubei 430081 China; 3 Chengdu Institute of Biology, Chinese Academy of Sciences, Chengdu, Sichuan 610041 China

## Abstract

Paired box 7 (*PAX7*) gene regulates the conversion of muscle satellite cells into myogenic cells and participates in multi-step processes in myogenesis. Expression levels of PAX7 are decisive for its regulatory function. Previous reports revealed that PAX7 were responsible for the developmental traits of muscle. The relationship of the *PAX7* promoter variants and livestock phenotypic traits has not been fully elucidated. We detected a novel 10-bp insertion/deletion (indel) polymorphism in the bovine *PAX7* promoter and revealed that the indel altered the binding of the transcriptional factor ZNF219. Luciferase reporter assay showed that deletion-deletion (Del-Del) genotype of the *PAX7* gene showed 2.79-fold higher promoter activity than the insertion-insertion (Ins-Ins) genotype (*P* < 0.05), and ZNF219 overexpression significantly diminished the luciferase activity in Ins-Ins groups. Moreover, the expression of *PAX7* and its down-stream genes were detected in fetal skeletal muscle of cattle with different *PAX7* genotypes, where the Del-Del genotype also displayed high expression levels. Statistical association analysis demonstrated that this indel had significant effects on early growth traits in cattle. These findings provide a complete overview of the function of the *PAX7* 10-bp variant, which may have potential as a genetic marker for marker-assisted selection in improving economically significant traits of cattle.

## Introduction

Cattle phenotypes of economic significance, such as growth rates, carcass, and meat quality, have attracted much attention from breeders in the beef cattle industry. Such phenotypes constitute a multifactorial background that arises from interactions between environmental and genetic factors^1^. In marker-assisted selection programs, the efficiency of selection is associated with the identification of candidate genes, as well as the exploration of genome-wide variation markers that are responsible for the complex quantitative traits^2^. The establishment of genetic association approach provides a unique opportunity to study genotype-phenotype relationships^3^. Therefore, the integration of screening for variations and gene association programs can improve assessments of the genetic effects of candidate genes, thus providing promising materials for successful genetic selection in cattle breeding.

Muscle satellite cells, known as muscle-specific committed progenitors, are located under the basal lamina of muscle fibres and play important roles in the growth, maintenance, homeostasis, repair, and regeneration of skeletal muscle^4–6^. Paired box 7 (*PAX7*) gene, a member of the paired box gene family, is the marker gene of myogenic satellite cells^7^. *PAX7* is specifically expressed in quiescent, activated, and proliferating satellite cells, and *PAX7*-expressing satellite cells are indispensable for the postnatal muscle development and regeneration after the injury of skeletal muscle, however, their regenerative function can not be performed by other endogenous cell types. In addition, the deficiency of the myogenic cells in skeletal muscle of the *PAX7* knockout mice demonstrated that the PAX7 was required for specification of the satellite cell lineage^8^. As a transcription factor, PAX7 affects the conversion of the myogenic progenitors entry into skeletal myoblast program by regulating the down-stream myogenic determination genes^9,10^. For example, Kumar *et al*.^11^ revealed that PAX7 can block premature differentiation and maintain stem cell status of quiescent satellite cells by inducing the expression of the inhibitor of DNA binding 2 (ID2) and ID3. Previous studies reported that *PAX7* knockout mice exhibited muscle malformations, and the body sizes of the homozygous deletion mice were significantly smaller as compared with the wildtype and heterozygous counterparts^12,13^. Therefore, given its fundamental roles in satellite cell differentiation and muscle development, we hypothesized that the *PAX7* could be considered as a selection marker gene for muscle involving phenotypic traits.

Previous association studies have identified substantial genetic variations in the coding regions of candidate genes that are responsible for economical traits of cattle^14–16^. In addition, regulatory promoter elements contribute to the promotion and suppression of gene transcription, thus, the exploration of the variants in the promoter region is also of the utmost importance. Promoter polymorphisms may change the binding sites of transcriptional factors that could completely alter the inducibility of the promoter^17^ or significantly influence the transcriptional activity^18^. The promoter sequence of *PAX7* comprises multiple regulatory elements^19^. In humans, a (CCT)_n_ microsatellite polymorphism, located in the specificity protein 1 (SP1) binding site, was detected in the *PAX7* promoter, and the (CCT)_11_ variant showed significantly higher transcriptional efficiency in comparison to the (CCT)_8_ and (CCT)_10_ genotypes^20^. Similarly, a G/C single nucleotide polymorphism (SNP) and a (CA)_n_ microsatellite variant have been identified in the promoter region of *PAX3* gene (a paralogue of *PAX7* gene)^21^. Our previous studies have reported that seven SNPs in the exon and intron regions of *PAX7* were significantly associated with growth traits of Chinese cattle breeds^22,23^. However, to our knowledge, there have been no reports about the *PAX7* promoter polymorphisms and their functional effects on economically significant traits in cattle.

The bovine *PAX7* gene is located on chromosome 2 and underlies the quantitative trait locus (QTL) for body weight^24^. The objectives of this study were to detect genetic polymorphism in the *PAX7* promoter among five Chinese indigenous cattle breeds, determine the relationship of the variation with growth traits, and further explore the biological effects of the *PAX7* gene with different allelic variants. These results may provide evidence for further investigation on exploiting the significant polymorphisms as molecular markers in cattle breeding programs.

## Results

### Identification of a novel *PAX7* 10-bp indel polymorphism

**Figure 1:**
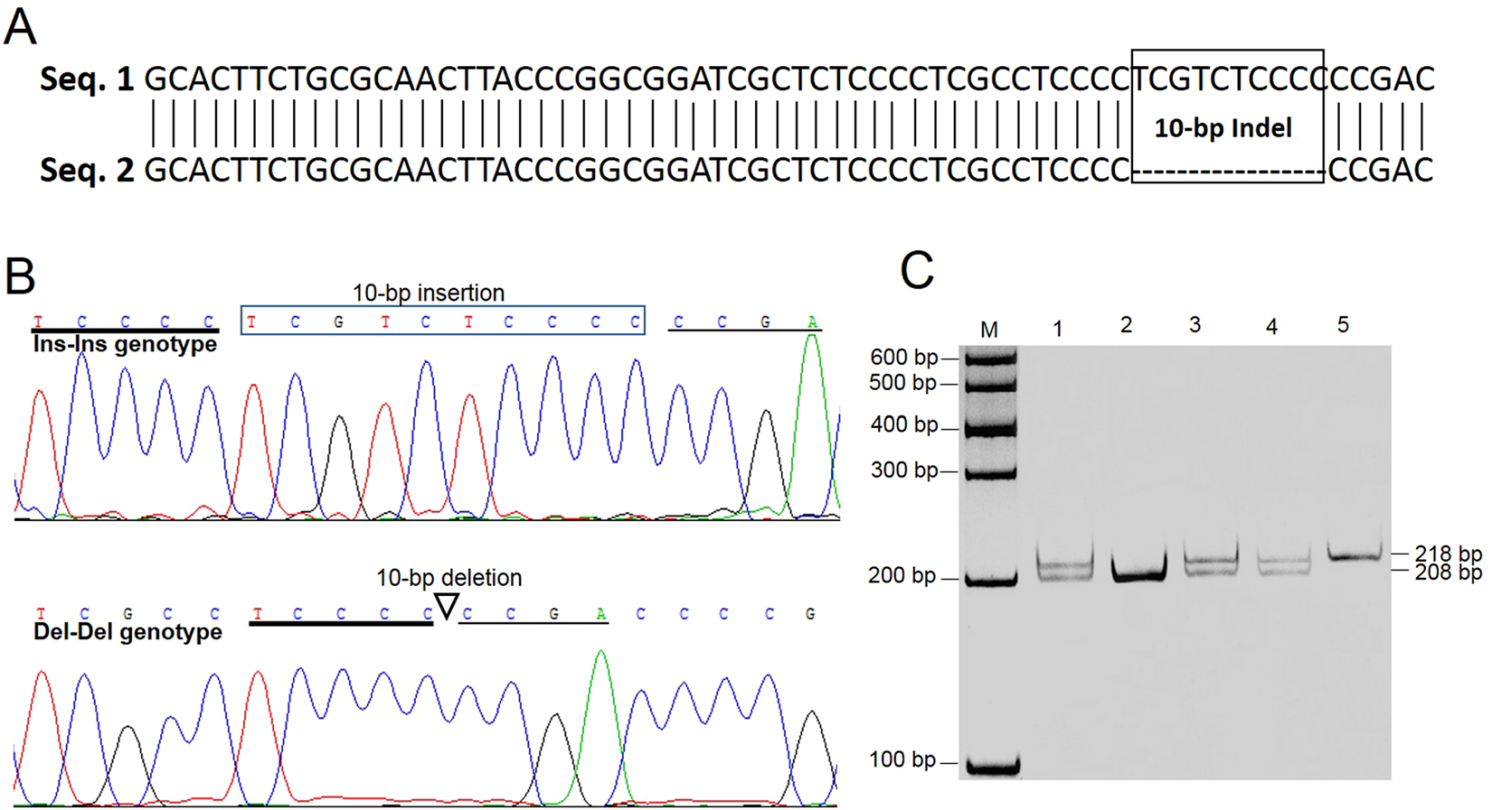
Identification of the 10-bp indel polymorphism in the bovine *PAX7* gene. (**A**) BLAST results for two different partial promoter sequences of *PAX7*. Seq. 1 was the sequences from NCBI (accession no. NC_007300); Seq. 2 was obtained by DNA sequencing of the bovine *PAX7* promoter with the 10-bp deletion. (**B**) Sequencing maps of the 10-bp indel polymorphism. Top: the rectangular frame contains the inserted nucleotides; bottom: the triangle shows the position of the 10-bp deletion. (**C**) Genotyping patterns of the 10-bp indel polymorphism obtained by polyacrylamide gels electrophoresis. Lanes 1, 3, 4: Ins-Del genotype; lane 2: Del-Del genotype; lane 5: Ins-Ins genotype.

We sequenced the 1868-bp promoter region of the bovine *PAX7* gene and revealed a 10-bp (TCGTCTCCCC) indel polymorphism between nucleotide position −633 and −643 (Fig. 1A,B). Using the P2 primer (Supplementary Table S1), the 10-bp indel variant was genotyped by polyacrylamide gel electrophoresis (PAGE) in five Chinese cattle breeds. As illustrated in Fig. 1C, the genotypic patterns were determined with a 208-bp fragment for the Del-Del genotype, a 218-bp fragment for the Ins-Ins genotype, and 208-bp and 218-bp fragments for the Ins-Del genotype. This polymorphic sequence has been deposited in the National Center for Biotechnology Information (NCBI accession number: ss 831883063).

### Genetic parameter analysis of the indel in five cattle breeds

Distributions of genotypic and allelic frequencies of the 10-bp indel, as well as its genetic diversity, were given in Table 1. The Del was the predominant allele in Nanyang (NY) breed, however, the Ins allele was predominant in Jiaxian (JX), Qinchuan (QC), Luxi (LX), and Chinese Caoyuan CY) populations. Interestingly, the results of a χ^2^ test showed that the 10-bp indel was in Hardy-Weinberg equilibrium (*P* > 0.05) in the NY breed, whereas the deviations from the Hardy-Weinberg equilibrium (*P* < 0.01) were detected in JX, QC, LX, and CY populations, which may be attributed to the selection and the population history, specifically, the degree of selection, small population size, and population mixture. The values of genetic heterozygosity (He) and effective allele numbers (Ne) were 0.460–0.550 and 1.853–2.000, respectively. According to the classification of polymorphism information content (PIC), all of the five cattle breeds exhibited moderate polymorphism (0.250 < *PIC* < 0.500) at the 10-bp indel locus, suggesting that the five cattle breeds may undergo the similarly continued selection pressure in evolutionary history.

**Table 1:** Genetic parameters of the *PAX7* 10-bp indel polymorphism in five cattle breeds.

Breeds	Sex	Genotypic frequency			Allelic frequency		He	Ne	PIC	HWE (*P*)^1^
		Ins-Ins	Ins-Del	Del-Del	Ins	Del				
NY (220)	Female	0.245	0.505	0.250	0.498	0.502	0.500	2.000	0.375	> 0.05^a^
JX (395)	Female	0.400	0.215	0.385	0.508	0.492	0.500	2.000	0.375	< 0.01
QC (224)	Female	0.513	0.254	0.232	0.641	0.359	0.460	1.853	0.354	< 0.01
LX (161)	Female	0.317	0.385	0.298	0.509	0.491	0.500	1.999	0.375	< 0.01
CY (233)	Female	0.468	0.129	0.403	0.532	0.468	0.498	1.992	0.374	< 0.01

^1^*P*-value with “a” indicates that the breed was in Hardy-Weinberg equilibrium (HWE). He, heterozygosity. Ne, effective allele numbers. PIC, polymorphism information content.

### Association analysis of the indel with cattle growth traits

As shown in Table 2, the 10-bp indel of the *PAX7* gene revealed significant associations with body weight (*P* = 0.0004), body height (*P* = 0.0248), body length (*P* = 0.0043), heart girth (*P* = 0.0001), hucklebone width (*P* = 0.0128) and average daily gain (*P* = 0.0006) in NY cattle with an age of 6 months, where the Del-Del genotype showed higher values than the Ins-Ins and Ins-Del genotypes. Regarding the individuals aged 12 months old, this indel was associated with body weight (*P* = 0.014), body length (*P* = 0.0035), and heart girth (*P* = 0.0001). Consistently, the cattle with Del-Del genotype also had significantly improved traits in comparison to those with Ins-Ins and Ins-Del genotypes. However, no associations were found between the indel locus and growth traits of cattle with ages of 18 and 24 months (*P* > 0.05, Supplementary Table S2).

**Table 2:** Association analysis of the *PAX7* 10-bp indel with growth traits in NY cattle aged 6/12 months.

Age	Growth traits	*PAX7*-10-bp indel genotypes (Mean ± SE)			*P*-value
		Ins-Ins(n = 54)	Ins-Del(n = 111)	Del-Del(n = 55)	
6 months	Body weight, kg	144.00 ± 4.61^B^	159.24 ± 2.44^A,b^	167.48 ± 3.32^A,a^	0.0004
	Body height, cm	102.71 ± 1.31^b^	106.42 ± 0.69^a^	106.96 ± 0.94^a^	0.0248
	Body length, cm	101.14 ± 1.46^B^	106.06 ± 0.77^A^	107.07 ± 1.05^A^	0.0043
	Heart girth, cm	121.86 ± 1.78^B^	128.82 ± 0.94^A,b^	132.15 ± 1.28^A,a^	0.0001
	Hucklebone width, cm	17.64 ± 0.34^B^	18.21 ± 0.18^b^	18.87 ± 0.25^A,a^	0.0128
	Average daily gain, kg	0.64 ± 0.02^B^	0.72 ± 0.01^A^	0.76 ± 0.02^A^	0.0006
12 months	Body weight, kg	210.71 ± 6.04^B^	220.86 ± 3.19^b^	232.11 ± 4.35^A,a^	0.0140
	Body length, cm	112.07 ± 1.91^B,b^	116.88 ± 1.01^a^	120.22 ± 1.38^A^	0.0035
	Heart girth, cm	134.50 ± 1.89 ^C^	140.62 ± 1.00^B^	145.44 ± 1.36^A^	0.0001

Within the same row, values with different lowercase letters (a,b) differ significantly at *P* < 0.05, and those with different uppercase letters (A, B, C) differ significantly at *P* < 0.01.

### Influence of the indel on the binding of the zinc finger transcription factor 219 (ZNF219)

**Figure 2:**
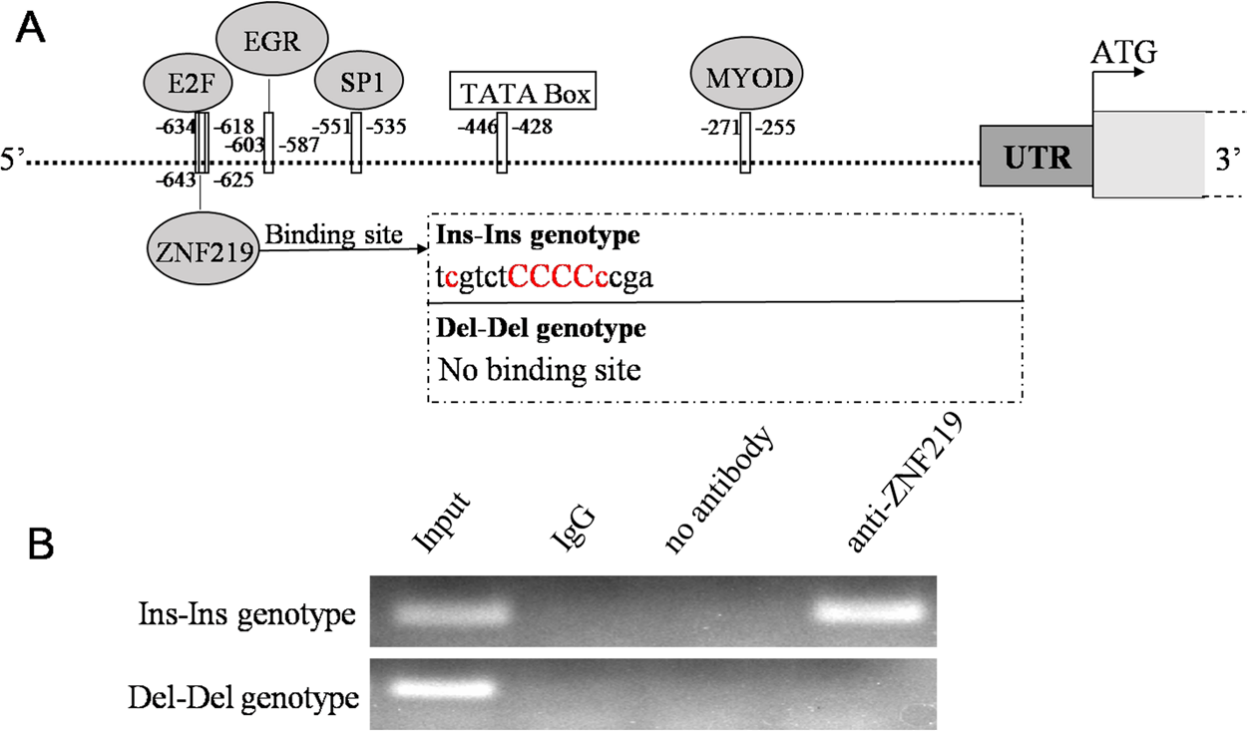
The *PAX7* 10-bp indel affects the binding site of ZNF219. (**A**) Schematic representation of the putative binding sites of TFs in the presence of Ins-Ins or Del-Del genotypes of the 10-bp indel polymorphism, as predicted by MatInspector Release professional 8.0.4. The sequence of the core binding site of ZNF219 transcription factor was shown, with ci-values of >60 in red and the core sequence in red capitals. Grey ellipses: predicted TFs; white box: TATA box element; dark-gray box: 5′-untranslated region (UTR); light gray box: coding region. (**B**) ChIP assay of ZNF219 binding to the *PAX7* gene promoter with Ins-Ins or Del-Del genotypes. The images were cropped from the same gel, and the full-length gel was shown in Supplementary Fig. S1.

Given the significant association between the *PAX7* 10-bp indel and cattle growth traits, the mechanism that contributed to these phenotypic variations remained to be determined. We hypothesized that the observed differences in genotype may reflect differences in the activity of the *PAX7* promoter. Therefore, the *PAX7* promoter region adjacent to the 10-bp indel was analyzed, and the results were shown in Fig. 2A. The binding sites of many transcriptional factors (TFs), such as myoblast determination protein (MYOD), SP1, early growth response (EGR), and E2F, were found in the analyzed sequences. Specifically, in the presence of the Ins-Ins genotype, the binding site of ZNF219 is created, while this site was lost in the presence of the Del-Del genotype (Fig. 2A). Correspondingly, previous studies have reported that ZNF219 functioned as a transcriptional regulator and was a sequence-specific DNA binding protein in nucleus^25^. In addition, we used chromatin immunoprecipitation (ChIP) assay to further test the binding of the ZNF219 to the 10-bp indel locus. The results indicated that ZNF219 bound to the Ins-Ins genotype of the *PAX7* promoter, while no binding was detected in the Del-Del genotype (Fig. 2B).

### Detection of the *PAX7* promoter activity

**Figure 3:**
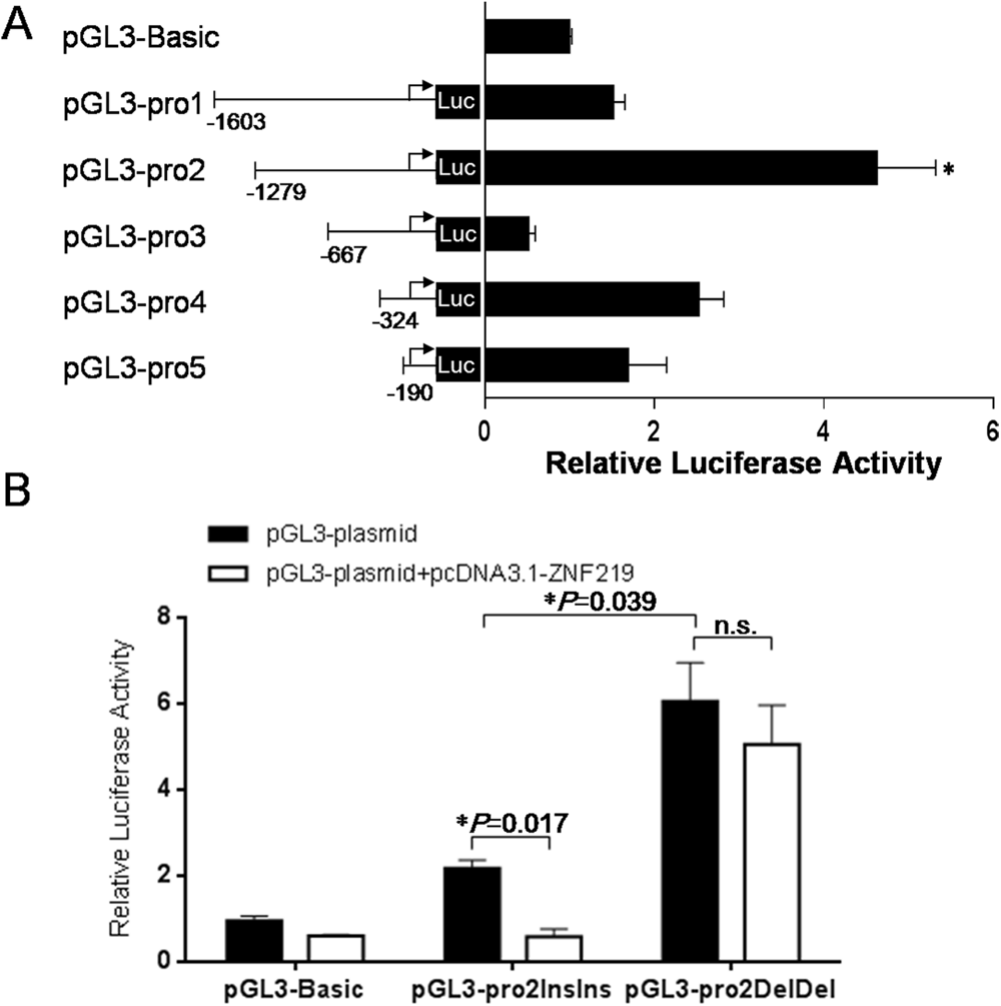
*PAX7* promoter activity modulated by the 10-bp indel polymorphism. (**A**) Luciferase activity in C2C12 cells transfected with recombinant plasmids containing serial promoter fragments (pGL3-pro1, pGL3-pro2, pGL3-pro3, pGL3-pro4, and pGL3-pro5) of the *Pax7* promoter. The backbone vector pGL3-Basic was used as a negative control. *P*_pGL3-pro2 vs. pGL3-pro1_ = 0.0118, *P*_pGL3-pro2 vs. pGL3-pro3_ = 0.0042, *P*_pGL3-pro2 vs. pGL3-pro4_ = 0.0495, *P*_pGL3-pro2 vs. pGL3-pro5_ = 0.0241. (**B**) Luciferase activity in C2C12 cells transfected with recombinant plasmids containing the Ins-Ins or Del-Del genotype. pcDNA3.1-ZNF219 was transiently co-transfected into C2C12 cells together with the reporter vectors. Each experiment was repeated at least three times. The data were mean ± standard error (S.E.) of the normalized luciferase activity. *P < 0.05 represents a significant difference.

In order to confirm whether the 10-bp indel was located in the core active region of the *PAX7* promoter, pGL3-pro1, pGL3-pro2, pGL3-pro3, pGL3-pro4, and pGL3-pro5 plasmids were co-transfected with pRL-TK into C2C12 cells, respectively. The results showed that the pGL3-pro2 vector yielded a significantly stronger fluorescence than the other vectors (*P* < 0.01 or *P* < 0.05 Fig. 3A). Notably, the pGL3-pro1 construct, which contained a larger portion of the *PAX7* promoter, displayed significantly lower promoter activity than the pGL3-pro2 construct (*P* < 0.05), suggesting that there may be an inhibitor binding site existing in the promoter region −1603~−1279 of the *PAX7* gene. In fact, the 10-bp Ins-Ins genotype was included in the pGL3-pro2 plasmid, denoted as pGL3-pro2InsIns. Similarly, the vector pGL3-pro2DelDel (including 10-bp Del-Del genotype) was constructed, and its transcription activity was also detected in C2C12 cells. As shown in Fig. 3B, the Del-Del genotype showed 2.79-fold higher luciferase activity than the Ins-Ins genotype (*P* < 0.05). Luciferase reporter assay was performed to determine whether ZNF219 regulated *PAX7* promoter activity (Fig. 3B). The results demonstrated that overexpression of ZNF219 significantly diminished the luciferase activity in the Ins-Ins groups (*P* < 0.05), while ZNF219 overexpression had no effects on the promoter activity in the Del-Del groups (*P* > 0.05).

### Influence of the indel on gene expression in the muscle of cattle

**Figure 4:**
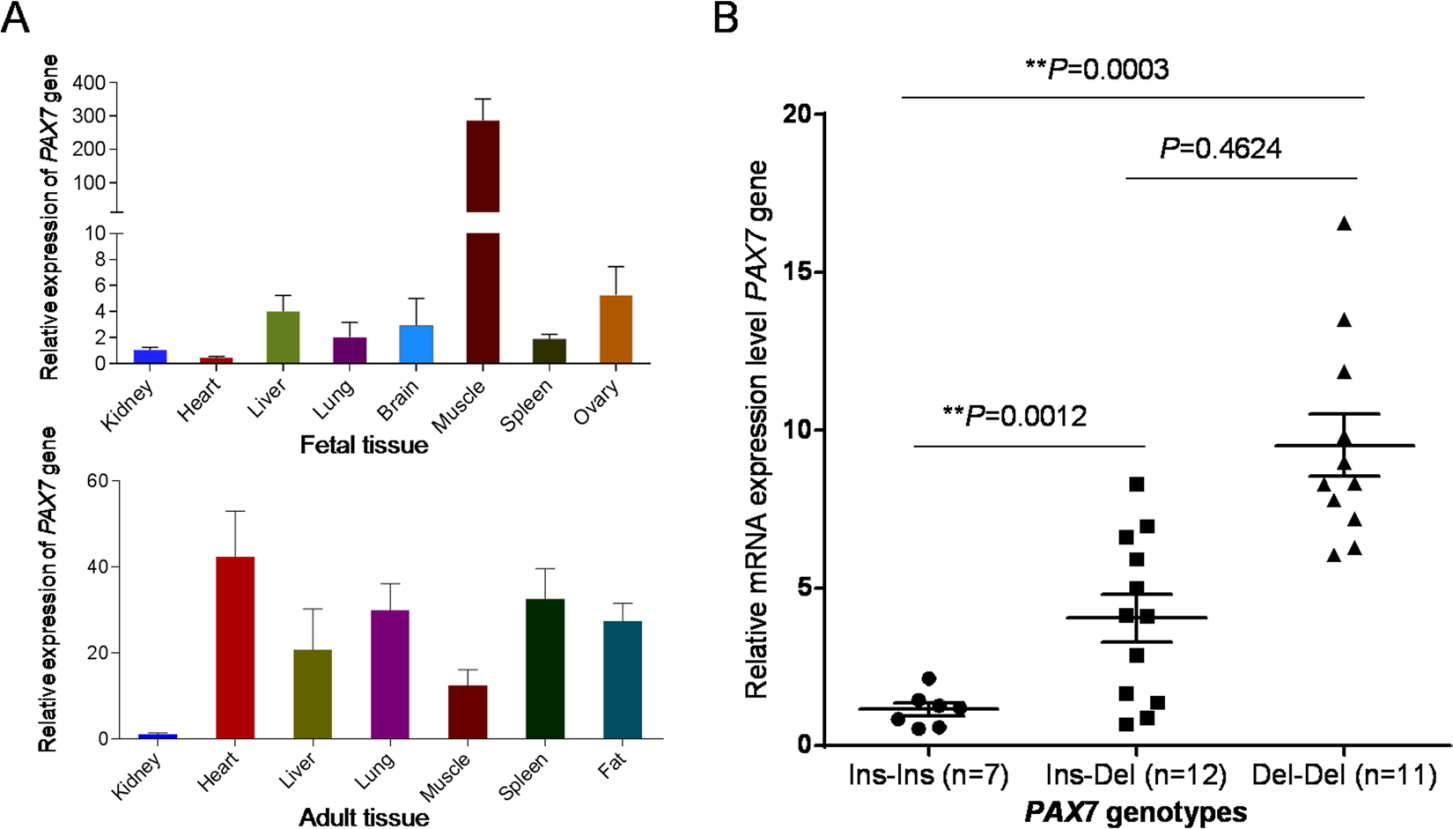
Expression levels of the *PAX7* gene in tissues of QC cattle. (**A**) Expression profiling of the *PAX7* gene in fetal and adult tissues. (**B**) Expression levels of the *PAX7* gene in skeletal muscles in cattle embryo with different genotypes. The relative expression levels of *PAX7* normalized that of bovine glyceraldehyde-3-phosphate dehydrogenase (*GAPDH*) are shown for all animals with the genotypes Ins-Ins (n = 7), Ins-Del (n = 12), and Del-Del (n = 11). The data are mean ± S.E. for each genotype group. **P < 0.01 represents a highly significant difference.

**Figure 5:**
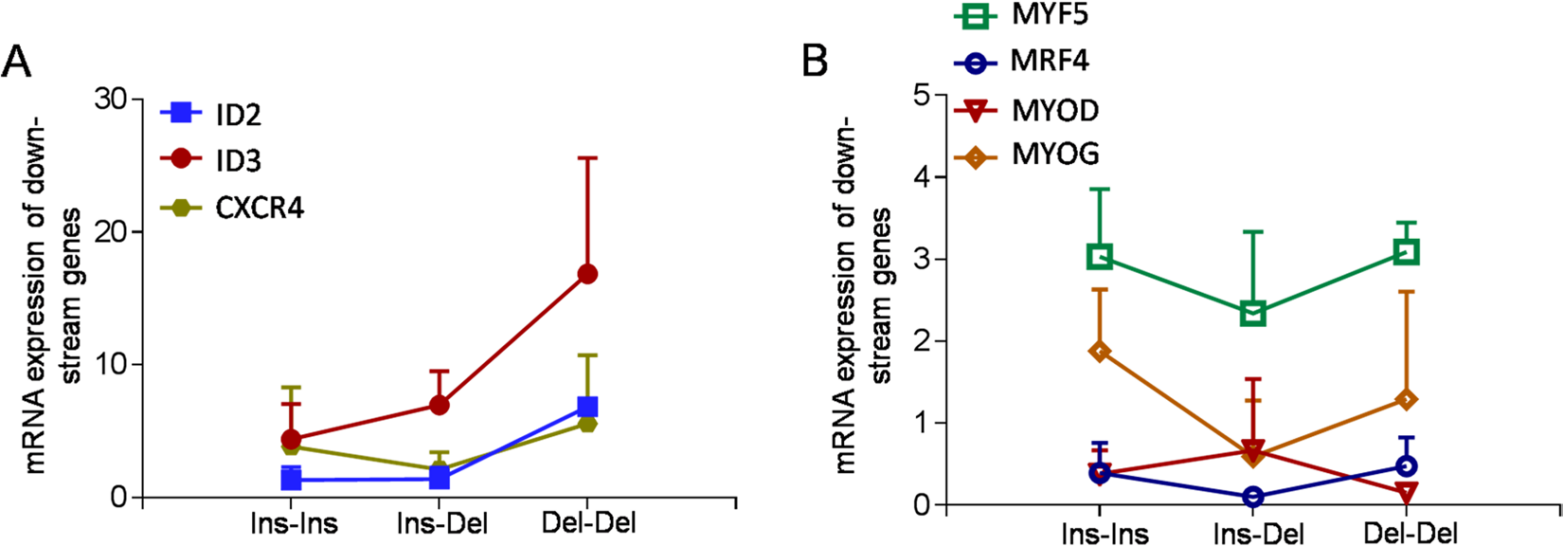
Expression patterns of the *PAX7* down-stream genes in fetal skeletal muscles of cattle with different genotypes. (**A**) Relative expression levels of genes expressed in myogenic satellite cells: *ID2*, *ID3*, and *CXCR4*. (**B**) Relative expression levels of genes involved in myogenic determination and differentiation: *MYF5*, *MRF4*, *MYOD*, and *MYOG*. The mRNA expressions were normalized to *GAPDH*. Each experiment was repeated at least three times. The data are the mean ± S.E. for each genotype group.

Expression pattern analysis revealed that the *PAX7* gene was highly expressed in the skeletal muscle of cattle embryos, whereas it displayed lower expression levels in other tissues (Fig. 4A). Thus, we collected the muscle tissues from 30 cattle embryos, in which 7, 12, and 11 individuals were genotyped as Ins-Ins, Ins-Del, and Del-Del, respectively. Next we detected the *PAX7* expression in the skeletal muscle of fetal cattle with different genotypes, and found that the Del-Del genotype showed 8.30-fold higher expression than the Ins-Ins group (*P* < 0.01, Fig. 4B). In addition, the expression of the *PAX7* down-stream genes were also investigated in the different genotypic groups. As shown in Fig. 5A, we found the same tendency of the higher expression of *ID2*, *ID3* and chemokine receptor 4 (*CXCR4*) in the individuals with the Del-Del genotype than the Ins-Ins and Ins-Del groups, which was in parallel with enhancive *PAX7* expression. However, the other four genes, myogenic factor 5 (*MYF5*), myogenic regulatory factor 4 (*MRF4*), myoblast determination protein (*MYOD*) and myogenin (*MYOG*), displayed non-significant differences among the three genotypic individuals (Fig. 5B).

## Discussion

The identification of DNA markers that contributes to phenotypic evolution is a powerful aid to animal breeding, importantly, thorough functional research of the causative markers should be explored before their application in breeding projects^26^. In cattle, previous studies have identified substantial indel variants acrossing the whole genome in different species^27,28^, however, there have been few attempts to associate indels with functional effects. In this study, we firstly detected a novel 10-bp indel variant in the promoter region of the *PAX7* gene in five Chinese cattle breeds, and further investigated the genetic diversity, characterization of genetic properties, and the determination of functional impacts.

Paired box (*PAX*) genes, termed as the *PAX* gene family, encode for specific DNA-binding transcription factors, which play critical roles in early development^29^. The *PAX* family includes nine genes that are assigned to four subgroups based on conservation of their primary structure: (1) *PAX1/PAX9*, (2) *PAX2/PAX5/PAX8*, (3) *PAX3/PAX7*, and (4) *PAX4/PAX6*^30^. *PAX3* and *PAX7* participate in the specification, survival, proliferation and self-renewal of muscle progenitor cells, and are required for skeletal muscle development^13^. The sequences of *PAX* genes are evolutionarily conserved among various species, suggesting that the genetic mutation may cause remarkable phenotypic alteration, even result in serious disease. For example, SNPs of the *PAX3* and *PAX7* have been associated with human alveolar rhabdomyosarcoma^31^. Our previous study reported that the SNPs in *PAX3* (promoter, exon and intron) and *PAX7* (exons and introns) showed significant associations with growth traits in Chinese cattle^22,23,32^. In the present study, we further detected the 10-bp indel in the *PAX7* promoter, and revealed that the indel significantly affect growth traits by regulating expression effects. Similarly, Zhang *et al*.^33^ found a novel 31-bp indel in intron 3 of the *PAX7* gene that was associated with chicken growth, carcass and meat quality traits. These results may be attributed to the location of the *PAX7* gene in QTL region that is linked to economic traits^24^.

The promoter, where multiple *cis*-transcriptional elements exist in, plays critical roles in modulating gene expression. Polymorphisms in the promoter region of a gene may affect its gene product by altering transcription factor binding sites or RNA stability^34^. Numerous studies have revealed that promoter variants can cause significantly potential phenotype diversity, and most of the regulatory mechanisms are associated with the change of transcription factor binding and promoter activity^35^. For example, a single mutation at position −1,687 in human *L-plastin* gene affected the binding strength of the transcriptional suppressor NKX3.1, and further reduced the expression of L-plastin and potentially decreased the tumorigenesis and progression of prostate cancer^36^. Dominquez *et al*. indicated that a 23-bp indel in porcine *TLR5* gene, creating an additional STAT binding site, is associated with an increase of the promoter activity^37^. We found a 10-bp indel variant in the promoter region of the bovine *PAX7* gene that the 10-bp insertion can reduce promoter activity and *PAX7* expression, and negatively associate the cattle growth traits by creating a transcriptional suppressor ZNF219 binding site, while the 10-bp deletion can enhance the effects. This 10-bp indel could be a potential selection marker for superior muscle involving traits in cattle breeding industry.

The results of promoter activity assay and association analysis demonstrated that ZNF219 negatively regulated the expression of the *PAX7* gene by binding to its promoter in the presence of the 10-bp indel. The *ZNF219* gene is a member of the Kruppel-like zinc finger gene family that are involved in many biological processes, such as cell growth, differentiation, embryogenesis and tumorigenesis, and ZNF219 is often depleted in the early stage of tumor progression^38^. Loss of ZNF219 expression is correlated with high oncogene level and the presence of some metastatic diseases^39^. There was evidence in the literature to support the claim that ZNF219 is a nuclear transcriptional factor that regulates the expression of its downstream target genes. For example, Sakai *et al*.^25^ reported that ZNF219 could modulate the expression of the *HMGN1* gene directly by binding its upstream DNA sequence. Recently, Liu *et al*.^40^ showed that the expression of ZNF219 was associated with skeletal muscle reduction in cancer cachexia. In our association analysis, the Ins-Ins genotype with the ZNF219 binding presented remarkably lower body weight, body height, body length, heart girth, hucklebone width and average daily gain than Del-Del genotype. This finding provided a foundation for further studies of the regulatory mechanisms and roles of ZNF219 on *PAX7* gene expression in the development of skeletal muscle.

Although it is not fully elucidated if the decreased *PAX7* expression is directly defined by the 10-bp insertion or is the result of interaction with other genetic pathways, it is interesting to note that the homozygous genotype (Ins-Ins) of the 10-bp indel, which reduced the promoter activity of *PAX7* by creating the ZNF219 binding site, was related to decreased phenotypic traits of cattle. Furthermore, we found that the significant associations of the 10-bp indel with growth traits were detected in the early stage (6 and 12 months old) of cattle. Consistently, the *PAX7* gene with Del-Del genotype showed higher expression than the Ins-Ins and Ins-Del genotypes in skeletal muscle of fetal cattle. These results may be attributed to the regulatory function of PAX7 in early muscle development. PAX7 participates in successive phases of embryonic and post-natal myogenesis that lead to the formation and growth of skeletal muscles^41^. Previous study reported that the body weight of *PAX7* knockout mice markedly decreased from birth to the age of two weeks, which was due to the absence of PAX7 in the progenitor satellite cells^12^. During myogenesis, PAX7 is considered as an upstream regulators that can regulate the satellite cells entry into the myogenic programme by stimulating transcriptional activation of target genes^42^. For instance, ID2 and ID3 are two notable PAX7 targets that are coordinately expressed with PAX7 in quiescent satellite cells and can be induced by ectopic expression of PAX7^11^. CXCR4 is another PAX7 target in satellite cells^43^. Herein, we found the same tendency of the high expression of *ID2*, *ID3* and *CXCR4* in the individuals with Del-Del genotype that was accompanied by PAX7 level. However, the expression of other four genes, *MYF5*, *MYOD*, *MRF4* and *MYOG*, were not associated with the genotypes of the *PAX7* 10-bp indel, which may be explained by the reason that the four genes play crucial roles in proliferation and differentiation of myoblast cells, especially, the *MRF4* and *MYOG* are expressed in a later stage of myogenesis^44^. Therefore, the *PAX7* 10-bp indel could be utilized in early selection project for cattle breeding.

In summary, we identified a 10-bp indel in the *PAX7* promoter and demonstrated that this indel, located in the ZNF219 binding site, affected the promoter activity and expression of the *PAX7* gene, which in turn was associated with phenotypic traits in the early stages of cattle. The regulatory mechanism of the 10-bp indel and the validation of the indel locus in other populations are need to be explored in further study. Taken together, this study revealed the functional role of the *PAX7* 10-bp indel and provided promising insight into its further exploitation in molecular breeding of cattle.

## Methods

### Ethics statement

The experiments and animal care were performed according to the Regulations for the Administration of Affairs Concerning Experimental Animals (Ministry of Science and Technology, China, 2004) and approved by the Institutional Animal Care and Use Committee (College of Animal Science and Technology, Northwest A&F University, China). Embryos of slaughtered cattle were collected from a local slaughterhouse, Tumen Abattoir (Xi’an, China). Cattle were allowed access to feed and water ad libitum under normal conditions. All animal experiments and methods were carried out in accordance with the approved guidelines and all efforts were made to minimize suffering.

### Animals and sample preparation

Blood samples were obtained from a multi-breed panel of up to 1233 adult individuals including five Chinese cattle breeds: NY (n = 220), JX (n = 395), QC (n = 224), LX (n = 161) and CY (n = 233). These breeds represent the main populations of P. R. China and are reared in the Henan, Henan, Shaanxi, Shandong and Jilin province, respectively. Calves were weaned at six months of age on average and raised from weaning to slaughter on a diet of corn and corn silage. Genomic DNA was isolated from blood samples according to the procedure used by Sambrock, J. & Russell, D. W.^45^. Records of growth traits in NY breed were collected for further statistical analysis, including body weight, body height, body length, heart girth, hucklebone width and average daily gain at different growth periods (6, 12, 18, and 24 months).

The muscle samples (taken from the longissimus thoracis) were collected from 30 cattle embryos (gestation 90 days) in the QC breed within 10 min after slaughter. Total RNA was prepared from the muscles with Trizol reagent (Takara, Japan) according to the manufacturer’s protocol. RNA integrity was monitored by denaturing 1% agarose gel electrophoresis. Concentrations and purities of RNA were measured by spectrophotometry. Reverse transcription into cDNA was performed using a PrimeScript^®^ RT reagent Kit (Perfect Real Time) (Takara, Japan) with 2 μg total RNA in a 20 μl reaction. Genomic DNA was extracted from the same muscles (10 mg) by two rounds of proteinase K digestion and phenol-chloroform extraction.

### DNA pool sequencing and genotyping

DNA pool sequencing has been explored as an efficient strategy to increase the detection throughput of SNPs and small indels. In this study, five DNA pools were constructed and each pool contained 80–100 individuals that were randomly chosen from the five cattle breeds, respectively. All DNA samples was dissolved to 50 ng/μl, and then contributed same volume to their respective pool^46^.

Based on the sequence of the bovine *PAX7* gene (GenBank accession no. NC_007300), the primer P1 (Table S1) was designed to amplify a 1868-bp fragment, which encompassed the bovine *PAX7* promoter region and a part of the exon 1. PCR amplification was carried out using the genomic DNA pool as template, and PCR product was sequenced on an automated sequencer (ABI PRISM 3730 DNA analyzer). Sequence polymorphisms were identified *in silico* using BLASTN and sequencing maps results. In fact, a 10-bp insertion or deletion (10-bp indel) was identified at the position of −633 and −643 of the *PAX7* promoter region. Correspondingly, another primer pair P2 (Table S1) was designed for genotyping the 10-bp indel variant. The different genotypic fragments were separated on 10% PAGE in 1 × TBE buffer with ethidium bromide staining.

### Analysis of transcription factor binding sites in *PAX7* promoter region

The bovine *PAX7* promoter sequence was analyzed to identify putative regulatory regions *in silico* using TFSEARCH (www.cbrc.jp/research /db/TFSEARCH.html) and Genomatix MatInspector Release professional 8.0.4, to make sequence-based prediction of TF binding sites^47^.

### Chromatin immunoprecipitation (ChIP) assay

ChIP assay was strictly operated according the protocol of SimpleChIP Enzymatic Chromatin IP kit (Cell Signaling Technology). Cells were fixed with 1% formaldehyde for 10 min to cross-link DNA-protein complexes and quenched with glycine for 5 min. After washing with ice-cold PBS for three times, cells were re-suspended in lysis buffer. Then the lysis was digested by micrococcal nuclease at 37 °C for 20 min to length of approximately 150–900 bp. Digestion was terminated by the addition of 0.5 M EDTA. Chromatin was sonicated at 30% output for 6 × 10 sec. Clarify lysates by centrifugation at 10,000 rpm for 10 min at 4 °C. The supernatant was diluted with ChIP buffer (1:9). Add the immunoprecipitating anti-ZNF219 antibody (Atlas antibodies) into 500 μl of the diluted chromatin. IgG (Santa cruz) was used as the negative control. Reverse cross-linked DNA was purified by spin columns. The DNA was used as a template for PCR, and the products were separated on 1% agarose gel. The *PAX7* promoter region primers: Forward: 5′- GTTACAACCAGCACTTCTGC -3′, Reverse: 5′- TCTGGGGAGGGAAGAAGGAA -3′.

### Plasmid construction

According to the TF binding sites prediction results, five fragments of the *PAX7* promoter region were amplified by PCR using different primer pairs (Table S1). The large 1671-bp fragment spanning −1603 to +60, was cloned into the pGEM-T vector (Promega, Madison, WI). For luciferase reporter construction, the insert was released by *Nhe*I and *Hin*dIII digestion, and then subcloned into the luciferase reporter vector pGL3-Basic (Promega, Madison, WI). This plasmid was designated as pGL3-pro1. Serial deletion vectors, pGL3-pro2 (−1279 to +60), pGL3-pro3 (−667 to +60), pGL3-pro4 (−324 to +60), pGL3-pro5 (−190 to +60) were constructed from pGL3-pro1 using the primers including *Kpn*I and *Hin*dIII recognized sites, respectively. Additionally, the two vectors, termed as pGL3-pro2InsIns (10-bp Ins-Ins genotype) and pGL3-pro2DelDel (10-bp Del-Del genotype), were constructed for detecting the effects of the *PAX7* indel. Their inserted sequences were obtained from the individuals with homozygous locus. The vector pRL-TK (Promega, Madison, WI) was used as internal reference in the luciferase reporter system.

To generate *ZNF219* overexpressing vector, the full length of *ZNF219* coding sequence was amplified using a pair of specific primer with *Bam*HI and *Eco*RI, respectively (Table S1), and were cloned into the plasmid pcDNA3.1( + ). All recombinants were verified by DNA sequencing to proof correct insertion and proper orientation.

### Cell culture

A skeletal muscle cell line of C2C12 cells was grown in Dulbecco’s modified Eagle’s medium (DMEM) supplemented with 10% fetal bovine serum (Invitrogen, Carlsbad, CA), and 100 units/ml penicillin with 100 μg/ml streptomycin at 37 °C in a humidified 5% CO_2_ atmosphere.

### Transient transfection and luciferase reporter assay

Transfections were conducted using Lipofectamine 3000 (Invitrogen, Carlsbad, CA) according to the manufacturer’s protocol. Before transfection, C2C12 cells were seeded into 48-well plate at a density of 2 × 10^4^ cells/well in 1 ml of complete medium and cultured overnight. Luciferase reporter constructs containing the *PAX7* promoter with 10-bp insertion (pGL3-pro2InsIns) or 10-bp deletion (pGL3-pro2DelDel) sites and/or pcDNA3.1-ZNF219 plasmid were temporarily transfected into C2C12 cells using Lipofectamine 3000. To normalize the transfection efficiency, 40 ng of the pRL-TK Renilla transfection control plasmid was co-transfected into the cells. After 48 hr of serum starvation, the cells were lysed, and the luciferase activity was measured using the Dual Luciferase Assay System (Promega, Heidelberg, Germany) and the SpectraMax M5 reader (Molecular Devices, CA). The luciferase signal from the *PAX7* promoter reporter constructs were calculated and normalized to the Renilla luciferase activity^48^. All transfections were carried out in triplicate.

### Quantitative real-time PCR

Quantitative real-time PCR (qPCR) was performed to detect the expression of the *PAX7* and its down-stream genes on a Bio-Rad CFX 96^TM^ Real Time Detection System (Bio-Rad, Hercules, CA). The bovine glyceraldehyde-3-phosphate dehydrogenase (*GAPDH*) gene was used as an endogenous control to normalize the differences in the amount of total cDNA added to each reaction. Gene-specific qPCR primer pairs were designed using the Beacon Designer^TM^ software (version 8.1) (Table S1). The reaction contained 100 ng of cDNA, 10 μl SYBR^®^ Premix Ex Taq TM II (TaKaRa, Japan), and 10 pmol of primers in a volume totaling 20 μl. The mixture was denatured for 30 s at 95 °C and was followed by 40 cycles of 5 s at 95 °C and 30 s at 60 °C.

### Statistical analysis

The genotypic and allelic frequencies of the 10-bp indel within the *PAX7* gene were estimated by the standard counting method. Each breed and the overall dataset were tested for deviation from Hardy-Weinberg equilibrium by χ^2^-test performed by the POPGENE software^23^ (version 3.2). PIC, He, and Ne were determined using the methods of Nei and Roychoudhury^49^. The association analysis with the growth traits was established using the General Linear Model (GLM) procedure as implemented in the SPSS software^50^ (version 18.0). The traits and *PAX7* genotypes were statistically analyzed according to our previously reported statistical model^23^: *Y*_*ijkl*_ = *μ + BF*_*i*_ + *A*_*j*_ + *G*_*k*_ + *e*_*ijkl*_, where *Y*_*ijkl*_ is the observation of the growth traits; *μ* is the overall mean of each trait, *BF*_*i*_ is the fixed effect associated with *i*th breed and farm, *A*_*j*_ is the effect due to *j*th age, *G*_*k*_ is the fixed effect of *k*th genotype of the 10-bp indel and *e*_*ijkl*_ is the random residual error.

Where appropriate, data were expressed as mean value ± standard error of duplicates. For comparison of the mean of two groups, statistical significance was assessed by Student’s *t*-test^51^. Calculations were performed with the STATA Statistical Software (StataCorp, College Station, TX, USA). Statistical significance was defined as *P* < 0.05.

## Electronic supplementary material

Supplementary Table S1

Supplementary Table S2

Supplementary Figure S1
