# Supplementary material for: A novel PAX7 10-bp indel variant modulates promoter activity, gene expression and contributes to different phenotypes of Chinese cattle
Source: Sci Rep. 2018 Jan 29;8:1724. doi: 10.1038/s41598-018-20177-8 (PMC5789009; doi:10.1038/s41598-018-20177-8)
Supplement: Supplementary file 1 — Supplementary Table S1 [file 41598_2018_20177_MOESM1_ESM.pdf]

Title page

A novel *PAX7* 10-bp indel variant modulates promoter activity, gene expression and contributes to different phenotypes of Chinese cattle

Yao Xu[1,2], Tao Shi[1], Yang Zhou[1], Mei Liu[1], Sebastian Klaus[3], Xianyong Lan[1], Chuzhao Lei[1], Hong Chen[1 *]

[1] College of Animal Science and Technology, Northwest A & F University, Shaanxi Key Laboratory of Molecular Biology for Agriculture, Yangling, Shaanxi 712100, China

[2] Institute of Biology and Medicine, College of Life Science and Health, Wuhan University of Science and Technology, Wuhan, Hubei 430081, China

[3] Chengdu Institute of Biology, Chinese Academy of Sciences, Chengdu, Sichuan 610041, China

*Corresponding Author: Hong Chen

Tel: +86-029-87092012.

Fax: +86-029-87092164.

E-mail: chenhong1212@263.net

Address: No.22 Xinong Road,
College of Animal Science and Technology,
Northwest A&F University,
Yangling, Shaanxi 712100, P. R. China.

Supplementary Table S1 Information of primer sets used in this study.

Primer name	Primer sequences (5'-3')	Annealing temperature (^{o}C)	Fragment size (bp)
P1	F: GCAGAAGGGTCCGGTCATTC<br>R: ACCTTCCAGGGGAAACCCC	61.6	1868
P2	F: CGCGTTACAACCAGCACTTC<br>R: GCCTTCTTTCTCCGACCAC	59.2	208/218
pGL3-pro1	F: CTA***GCTAGC***AAATGGGGCTCCCTCCT<br>R: CT***AAGCTT***AAAACCGCTTTCCGTCC	60.5	1680
pGL3-pro2	F: GT***GGTACC***CGGGCAAGAACAGGACA<br>R: CT***AAGCTT***AAAACCGCTTTCCGTCC	66.3	1355
pGL3-pro3	F: GT***GGTACC***CTTACCCGGCGGATCGCTCT<br>R: CT***AAGCTT***AAAACCGCTTTCCGTCC	61.7	743
pGL3-pro4	F: GT***GGTACC***CCCGATCCCCCCCGCCA<br>R: CT***AAGCTT***AAAACCGCTTTCCGTCC	68.9	400
pGL3-pro5	F: GT***GGTACC***TGTTTGTTTGAACTTCCTC<br>R: CT***AAGCTT***AAAACCGCTTTCCGTCC	64.0	266
pEGFP-ZNF219	F: G***GAATTC***TATGGAGGGCTCACGTCCC<br>R: G***GGATCC***CTACCTTTCTTGGCCCCCT	66.2	2166
pcDNA3.1-ZNF219	F: G***GGATCC***ATGGAGGGCTCACGTCCC<br>R: G***GAATTC***CTACCTTTCTTGGCCCCCT	66.2	2166
qPCR primer			
Pax7	F: GAGGACGAAGCGGACAAGAA<br>R: TACGCTTCAGAGGGAGGTCG	60	139
Id2	F: ATCGTTAGCCTGCACCACC<br>R: TCAGCCACAGAGAGCTTTGC	60	153
Id3	F: CGCGTCATCGACTACATCCT<br>R: GATCACAAGTTCCGGAGCGA	60	117
CXCR4	F: TCTCCTGCCTGGTATCGTCA<br>R: AATGTAGTAGGGCAGCCAGC	60	148
Myf5	F: CCCACCTCAAGTTGCTCTGA<br>R: AGGAGCTTTTATCCGTGGCA	60	127
MRF4	F: GGCTGGATCAGCAGGACAAA<br>R: CAGTTATCACGAGCCCCCTG	60	153
MyoD	F: GAACACTACAGCGGCGACTC<br>R: GCTGTAGTAAGTGCGGTCGT	60	126
MyoG	F: GCAGCGCCATCCAGTACATA<br>R: GAGCTGCATTCACTGGGCA	60	124
GAPDH	F: AATGAAAGGGCCATCACCATC<br>R: GTGGTTCACGCCCATCACA	60	204

The italic and bold bases indicate recognition sites of restriction endonucleases, and the underlined were attached nucleotides
