# Supplementary material for: A novel PAX7 10-bp indel variant modulates promoter activity, gene expression and contributes to different phenotypes of Chinese cattle
Source: Sci Rep. 2018 Jan 29;8:1724. doi: 10.1038/s41598-018-20177-8 (PMC5789009; doi:10.1038/s41598-018-20177-8)
Supplement: Supplementary file 2 — Supplementary Table S2 [file 41598_2018_20177_MOESM2_ESM.pdf]

Title page

A novel *PAX7* 10-bp indel variant modulates promoter activity, gene expression and contributes to different phenotypes of Chinese cattle

Yao Xu[1,2], Tao Shi[1], Yang Zhou[1], Mei Liu[1], Sebastian Klaus[3], Xianyong Lan[1], Chuzhao Lei[1], Hong Chen[1 *]

[1] College of Animal Science and Technology, Northwest A & F University, Shaanxi Key Laboratory of Molecular Biology for Agriculture, Yangling, Shaanxi 712100, China

[2] Institute of Biology and Medicine, College of Life Science and Health, Wuhan University of Science and Technology, Wuhan, Hubei 430081, China

[3] Chengdu Institute of Biology, Chinese Academy of Sciences, Chengdu, Sichuan 610041, China

*Corresponding Author: Hong Chen

Tel: +86-029-87092012.

Fax: +86-029-87092164.

E-mail: chenhong1212@263.net

Address: No.22 Xinong Road,
College of Animal Science and Technology,
Northwest A&F University,
Yangling, Shaanxi 712100, P. R. China.

Supplementary Table S2 Association analysis of the *PAX7* 10-bp indel with growth traits in NY cattle aged 18/24 months.

Age	Growth traits	*PAX7*-10-bp indel genotypes (Mean ± SE)			*P*-value
		Ins-Ins(n = 54)	Ins-Del(n = 111)	Del-Del(n = 55)	
18 months	Body weight, kg	312.21±8.06	299.70±4.27	294.96±5.81	0.224
	Body height, cm	120.79±1.80	121.20±0.95	123.15±1.29	0.412
	Body length, cm	129.79±1.85	130.36±0.98	128.96±1.33	0.700
	Heart girth, cm	159.00±2.12	156.90 ±1.12	154.11±1.52	0.144
	Hucklebone width, cm	23.46±0.49	23.33±0.26	22.63±0.35	0.216
	Average daily gain, kg	0.46±0.06	0.44±0.03	0.35±0.04	0.118
24 months	Body weight, kg	358.86±10.02	373.45±5.35	366.74±7.21	0.408
	Body height, cm	125.29±1.13	127.22±0.60	126.44±0.81	0.301
	Body length, cm	136.29±1.99	139.06±1.07	138.25±1.44	0.475
	Heart girth, cm	166.36±2.65	170.00±1.42	168.57±1.91	0.466
	Hucklebone width, cm	25.00 ±0.64	25.43±0.34	25.28±0.46	0.837
	Average daily gain, kg	0.26±0.07	0.41±0.04	0.40±0.05	0.174
