# Supplementary material for: A novel PAX7 10-bp indel variant modulates promoter activity, gene expression and contributes to different phenotypes of Chinese cattle
Source: Sci Rep. 2018 Jan 29;8:1724. doi: 10.1038/s41598-018-20177-8 (PMC5789009; doi:10.1038/s41598-018-20177-8)

Title page

A novel *PAX7* 10-bp indel variant modulates promoter activity, gene expression and contributes to different phenotypes of Chinese cattle

Yao Xu[1,2], Tao Shi[1], Yang Zhou[1], Mei Liu[1], Sebastian Klaus[3], Xianyong Lan[1], Chuzhao Lei[1], Hong Chen[1 *]

[1] College of Animal Science and Technology, Northwest A & F University, Shaanxi Key Laboratory of Molecular Biology for Agriculture, Yangling, Shaanxi 712100, China

[2] Institute of Biology and Medicine, College of Life Science and Health, Wuhan University of Science and Technology, Wuhan, Hubei 430081, China

[3] Chengdu Institute of Biology, Chinese Academy of Sciences, Chengdu, Sichuan 610041, China

*Corresponding Author: Hong Chen

Tel: +86-029-87092012.

Fax: +86-029-87092164.

E-mail: chenhong1212@263.net

Address: No.22 Xinong Road,
College of Animal Science and Technology,
Northwest A&F University,
Yangling, Shaanxi 712100, P. R. China.

Supplementary Figure S1 Full-length gel of ZNF219 binding to the *PAX7* gene promoter with Ins-Ins or Del-Del genotypes by ChIP assay.

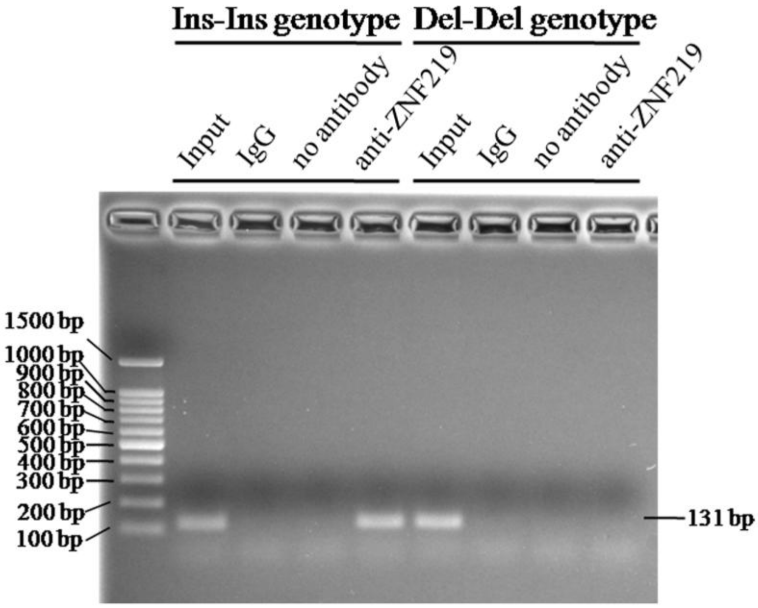

Supplement: Supplementary file 3 — Supplementary Figure S1 [file 41598_2018_20177_MOESM3_ESM.pdf]
